# Early Outcome of Elective and Emergent Endovascular Aortic Repair with the Minos^TM^ Abdominal Aortic Stent-Graft

**DOI:** 10.3390/jcm15114229

**Published:** 2026-05-30

**Authors:** Julia Caroline Krüger, Anna-Leonie Menges, Viktoria Pöll, Benedikt Reutersberg, Alexander Zimmermann

**Affiliations:** Department of Vascular Surgery, University Hospital Zurich, 8091 Zurich, Switzerland; julia.krueger@usz.ch (J.C.K.); anna-leonie.menges@usz.ch (A.-L.M.); viktoria.poell@usz.ch (V.P.); benedikt.reutersberg@usz.ch (B.R.)

**Keywords:** aortic aneurysm, EVAR, endoleak, low-profile stent-graft, sac regression

## Abstract

**Background/Objectives:** the study aims to evaluate early outcomes of endovascular aneurysm repair (EVAR) using the ultra-low-profile Minos^TM^ Abdominal Aortic Stent-Graft in elective and emergent infrarenal abdominal aortic aneurysm (AAA), and compare its performance with established EVAR devices. **Methods:** This retrospective single-centre study included 79 patients treated with the Minos stent-graft between September 2020 and August 2024. Primary endpoints were 30-day mortality and major adverse events (MAEs). Secondary outcomes included technical success, endoleak rates, sac dynamics, reintervention rates, and stent-graft patency. Imaging follow-up was performed on day 1, at 4–6 weeks, at 6–12 months, and annually thereafter. **Results:** The cohort (mean age 74.6 ± 8.2 years; 83.5% male) included 60 elective and 19 emergent cases, with high cardiovascular comorbidity and challenging anatomy (48.1% with iliac diameters < 7 mm and 16.5% < 5 mm; 20.3% hostile necks). Technical success rate was 93.7% (elective: 95.0%; emergent: 89.5%). Persistent endoleaks occurred in 14.0% of patients (type II 12.7%, type I 1.3%). Clinical success was achieved in 88.6% (elective: 90.0%; emergent: 84.2%). Six-month survival was 96.1% in the elective and 89.5% in the emergent cohort. MAEs were more frequent in emergent cases: acute kidney failure (31.6% vs. 3.3%; *p* = 0.004) and pneumonia (31.6% vs. 0.0%; *p* < 0.001). Mean follow-up was 12.5 ± 9.9 months (median 9.3). Reinterventions were required in 16.5% within 30 days, more frequently in emergent cases (31.6% vs. 11.7%, *p* = 0.041). Sac regression ≥5 mm occurred in 43.0%. No stent-graft migrations or infections were reported. **Conclusions:** Minos demonstrated reliable performance and safety in elective and emergent EVAR with excellent anatomical applicability due to its ultra-low-profile design.

## 1. Introduction

Abdominal aortic aneurysms (AAA) are common and potentially fatal, with rupture risk increasing with aneurysm size [1]. Once rupture occurs, mortality exceeds 50% [2,3], even with immediate intervention [4]. Elective repair is recommended for AAAs ≥5.5 cm in men (≥5.0 cm in women), symptomatic aneurysms, or those growing rapidly [5] to prevent rupture and related complications [6].

Endovascular aneurysm repair (EVAR) has become the preferred treatment for infrarenal AAAs in anatomically suitable patients, with lower perioperative morbidity and mortality than open surgery [7,8,9]. Its use has expanded to urgent and emergent cases, including symptomatic and ruptured AAAs [10], especially among elderly high-risk patients [11,12].

EVAR success depends on favourable anatomy, adequate sealing zones, and iliac access. Main bodies often require delivery systems of 18–20 French (Fr). This may be unsuitable for patients with narrow or calcified iliac arteries, which would necessitate surgical conduits and increase procedural risk. Therefore, low-profile stent-grafts, like the Gore Excluder (W. L. Gore & Associates, Inc., 32,360 N. North Valley Parkway, Phoenix, AZ 85085, USA) [13] and Cook Zenith Alpha (Cook Medical, 750 N Daniels Way, Bloomington, IN 47404, USA) [14] with 16 Fr, or even ultra-low-profile stent-grafts, like the Cordis Incraft (Cordis, 14,201 Northwest 60th Avenue, Miami Lakes, FL 33014, USA) [15] (14 Fr), have been developed. They feature reduced delivery-system diameters, and have demonstrated good outcomes in elective EVAR and acceptable results in selected cases of rupture. Still, some of those devices show high rates of limb graft occlusions or thromboembolic events [16,17].

The Minos^TM^ abdominal aortic stent-graft (hereafter referred to as Minos), CE-marked in 2019 (Lombard/Endovastec Limited, 4 Trident Park, Didcot, Oxfordshire, OX11 7HJ, United Kingdom) [18] is an ultra-low-profile trimodular endograft designed for infrarenal AAA repair, featuring a 14–16 Fr delivery system for the main body (CM) and 12 Fr for iliac limbs (CL), markedly reducing access requirements. Early European reports suggest high procedural success, low complication rates and favourable imaging outcomes in elective and emergent settings [19,20,21].

Despite promising initial data, clinical experience with Minos remains limited, and comparative studies with established devices are lacking. Most available reports focus on elective repair, but emergency use is not accounted for.

This study evaluates early outcomes of EVAR with the Minos stent-graft in elective and emergent settings and compares these findings with published data on other low-profile endografts within the context of the current literature.

## 2. Materials and Methods

### 2.1. Study Design

This single-centre, retrospective cohort study included all patients who underwent infrarenal EVAR with the Minos device (Microport, Oxfordshire, United Kingdom) between September 2020 and August 2024 and had provided institutional general consent for secondary use of their data, as required by the approval of the regional ethics committee. Eligible patients were adults (≥18 years) with infrarenal abdominal aortic aneurysms (fusiform, saccular, or penetrating aortic ulcers) treated electively or emergently (symptomatic or ruptured aneurysms). Treatment decisions were made by an interdisciplinary vascular board based on aneurysm morphology, patient-specific risk factors, and guideline recommendations.

In both elective and emergent cases, the Minos stent-graft was used as the standard endograft system at the institution, reflecting institutional practice and routine on-site availability, enabling timely treatment particularly in emergent settings. Device selection was not limited to specific anatomical subgroups or operator preference, and alternative treatment strategies would have been used if anatomical conditions had precluded its application. EVAR procedures were performed by a team of consultant vascular surgeons with extensive experience.

Elective indications included aneurysm diameter ≥ 5.5 cm in men, ≥5.0 cm in women or growth > 1 cm/year. Emergent EVAR was performed for contained or free rupture and severe aneurysm-related pain. Exclusion criteria were lack of research consent, use of physician-modified or adjunct fenestrated/branched techniques, mycotic aneurysms or connective tissue disorders.

### 2.2. Device Description

The Minos stent-graft is a trimodular endovascular device for infrarenal AAA repair, incorporating a low-profile delivery system: 14–16 Fr for the main body (CM) and 12 Fr for the iliac limbs (CL). The Y-shaped main body and the iliac limb components are introduced via bilateral femoral artery access and assembled in vivo at the target aortic segment. When required, a proximal cuff extension (CC) may be used to increase the proximal sealing zone. The stent-graft framework is manufactured from electropolished nitinol, while the graft material consists of seamless high-tenacity polyester multifilament fibres. Proximal fixation is achieved using eight laser-cut bare suprarenal fixation elements and is supported by a doubled proximal nitinol stent providing additional radial support.

The iliac limb components are available in various diameters and lengths and exhibit a helical configuration. Radiopaque markers facilitate intraoperative orientation. Deployment is performed using a two-stage pusher-based release mechanism, allowing controlled expansion with final positional adjustment prior to definitive suprarenal fixation.

### 2.3. Imaging and Anatomical Assessment

Preoperative Computed Tomography Angiography (CTA) was analysed using 3D reconstruction software (Endosize, Therenva, Version 3.2.1, 74F, Rue de Paris 35,000 Rennes, France) [22]. Proximal neck length was measured from the lowest renal artery to the onset of aneurysm sac (increase ≥10–15% in diameter). Aortic angulation was defined as the angle between the suprarenal and infrarenal centrelines (alpha angle), in accordance with the device instructions for use, and was used as a surrogate measure of proximal neck angulation relevant to device deployment and sealing.

Common iliac artery diameters and lengths were recorded, with the minimum luminal diameter noted to assess access feasibility. The iliac tortuosity index (TI; τ) was calculated as the ratio of the vessel centerline length between the common femoral artery and the aortic bifurcation (L1) to the corresponding straight-line distance between these two points (L2), expressed as τ = L1/L2. For analysis, TI was categorized into four groups, 0 (τ ≤ 1.25), 1 (1.25 < τ ≤ 1.5), 2 (1.5 < τ ≤ 1.6), and 3 (τ > 1.6), with higher categories reflecting increasing degrees of iliac tortuosity and anatomical complexity [23].

Aneurysm characteristics, including maximum sac diameter, thrombus burden, calcification, and manufacturer’s instructions for use (IFU) violations, were documented.

### 2.4. Procedural Details

All procedures were performed in a hybrid operating suite under general or regional anaesthesia. The Minos stent-graft was predominantly deployed within the anatomical IFU criteria, including a proximal neck length ≥ 15 mm and an angulation < 60°. In emergent cases or in frail patients unsuitable for open repair, deployment outside IFU was accepted. Oversizing of the main body and iliac limbs was approximately 15% and 10–20%, respectively. Transfemoral percutaneous access with closure devices was preferred using two Perclose ProStyle devices (Abbott Vascular, Baar, Switzerland) per femoral access site [24]; surgical cutdown was reserved for severe calcification. Completion angiography was performed in all cases to assess for endoleaks or technical issues. Additional procedure devices (e.g., proximal cuffs, endoanchors, stent-graft relining) were used at the surgeon’s discretion.

### 2.5. Follow-Up and Outcomes

Contrast-enhanced ultrasound (CEUS) was performed on postoperative day (POD) 1 as part of the institutional post-EVAR surveillance protocol to assess for early endoleaks in a standardized manner. In ruptured cases, CTA was performed immediately to assess stent-graft positioning and detect endoleaks after individual risk–benefit assessment, particularly considering the risk of contrast-induced nephropathy in patients with impaired renal function. Additionally, duplex ultrasound of access sites excluded access complications. Follow-up included CTA at 4–6 weeks to assess endoleaks, device-related issues, and sac size. In the absence of significant findings (Type I/III endoleak or sac enlargement), imaging was repeated at 6 months, then annually. Additional imaging (CTA or CEUS) was performed as needed. Clinical evaluations were accompanied by imaging, and follow-up data were collected up to a predefined study cut-off date in March 2025. Patients who missed scheduled visits were contacted, and clinical status was documented if imaging was declined.

### 2.6. Endpoints and Definitions

This study was guided by the 2021 Society for Vascular Surgery (SVS) reporting standards for complex aortic repair [25]. As the EVAR reporting standards date to 2002, the more recent framework was applied where appropriate.

Primary endpoints were 30-day and overall mortality and morbidity. Major adverse events (MAEs) included myocardial infarction (MI), pneumonia, stroke, spinal cord ischemia, clinically significant acute kidney injury (Stage 3 or requiring dialysis), bowel ischemia, sepsis, and limb occlusion (complete iliac limb thrombosis with loss of perfusion requiring intervention).

Secondary endpoints included technical and clinical success after EVAR. Technical success was defined as successful device introduction and deployment at the intended location, secure fixation, graft patency, and the absence of type I/III endoleaks at completion angiography. In accordance with established reporting standards, adjunctive intraoperative measures (e.g., additional modular components, stent implantation, or balloon angioplasty) performed to achieve a satisfactory final angiographic result did not preclude technical success. Access-related surgical procedures and perioperative complications were recorded separately.

Clinical success was defined as the absence of aneurysm-related mortality, persistent type I/III endoleaks, graft infection or thrombosis (≥25% circumference), sac expansion > 5 mm, aneurysm rupture, conversion to open repair during follow-up, device migration > 10 mm, permanent intervention-related major complications, such as paraplegia, disabling stroke or permanent dialysis (index or secondary intervention-related cause), and failure due to device integrity issues.

Hostile neck was defined according to established anatomical criteria, including proximal aortic neck length < 15 mm, angulation > 60°, or >50% circumferential thrombus or calcification, with the presence of any single criterion considered sufficient [26].

Anatomical off-IFU was defined as implantation of the Minos device outside the manufacturer’s instructions for use, including proximal neck length < 15 mm and angulation > 60°. Regulatory off-IFU was defined as off-label use in emergent or ruptured AAA cases, representing treatment outside the recommended IFU indications.

Aneurysm regression was defined as ≥5 mm sac diameter reduction compared to the preoperative scan.

Post-implantation syndrome was defined by fever ≥ 38 °C and leucocytosis (>12 × 10^9^/L), with or without elevated inflammatory markers, in the absence of infection after EVAR [27].

### 2.7. Statistical Analysis

Statistical analyses were conducted using IBM SPSS Statistics for Windows, Version 31.0.0.0 (IBM Switzerland Ltd., Zurich, Switzerland) [28]. Descriptive statistics included mean, standard deviation (SD), median, and range. Continuous variables were compared using two-tailed independent samples t-tests, and categorical variables using chi-square (χ^2^) tests. Survival outcomes were evaluated with Kaplan–Meier analysis, and group differences were assessed via the log-rank test. Statistical significance was defined as *p* < 0.05. Generative artificial intelligence (Microsoft 365 Copilot) was used during manuscript preparation to assist with text drafting and language refinement. The authors verified all content, and no AI tools were used for data analysis or interpretation.

## 3. Results

### 3.1. Patient Characteristics (Table 1)

Between September 2020 and August 2024, a total of 96 EVAR procedures were performed at the institution, all using the Minos stent-graft as the standard and routinely available endograft system. After applying predefined exclusion criteria, 17 patients were excluded (five declined consent; eight underwent fenestrated repair; and four had non-standard indications, including aorto-enteric fistulas or mycotic aneurysms), yielding a final cohort of 79 patients (60 elective, 19 emergent). The exclusions were not related to complications following Minos implantation but reflected differences in indication or treatment approach.

**Table 1:** Baseline characteristics and demographics among 79 patients treated with Minos stent-graft (elective: *n* = 60; emergent: *n* = 19).

Group	Total	Elective	Emergent	*p*-Value
	% (total) mean ± SD {median} [range]	% (total) mean ± SD {median} [range]	% (total) mean ± SD {median} [range]	
**Demographics**				
Age [years]	74.6 ± 8.2 {75.0} [60.0–89.0]	74.8 ± 7.1 {75.0} [61.0–88.0]	73.9 ± 11.2 {72.2} [60.0–89.0]	0.763
Sex				0.535
Men	83.5 (66/79)	85.0 (51/60)	78.9 (15/19)	
Women	16.5 (13/79)	15.0 (9/60)	21.1 (4/19)	
BMI [kg/m^2^]	26.5 ± 4.8 {26.2} [15.9–41.0]	26.1 ± 4.5 {25.4} [15.9–37.5]	28.0 ± 5.7 {27.5} [19.6–41.0]	0.136
**Medical history**				
CAD	43.0 (34/79)	45.0 (27/60)	36.8 (7/19)	0.531
MI	12.7 (10/79)	11.7 (7/60)	15.8 (3/19)	0.638
High cholesterol	32.9 (26/79)	31.7 (19/60)	36.8 (7/19)	0.676
Hypertension	72.2 (57/79)	71.7 (43/60)	73.7 (14/19)	0.864
Smoker ^a^	45.6 (36/79)	55.0 (33/60)	15.8 (3/19)	0.011
COPD	22.8 (18/79)	26.7 (16/60)	10.5 (2/19)	0.144
Diabetes	25.3 (20/79)	26.7 (16/60)	21.1 (4/19)	0.624
CKD	34.2 (27/79)	28.3 (17/60)	52.6 (10/19)	0.052
Baseline eGFR ^b^	66.6 ± 22.3 {63.0}	68.3 ± 19.7 {62.0}	61.3 ± 28.8 {63.0}	0.230
	[12.0–109.0]	[35.0–100.0]	[12.0–109.0]	
PAD	20.3 (16/79)	23.3 (14/60)	10.5 (2/19)	0.226
Stroke or TIA	11.4 (9/79)	6.7 (4/60)	26.3 (5/19)	0.019
Cancer	30.4 (24/79)	33.3 (20/60)	21.1 (4/19)	0.310
ACT ^c^	78.5 (62/79)	85.0 (51/60)	68.4 (13/19)	0.070
**ASA class**				<0.001
2	1.3 (1/79)	1.7 (1/60)	0.0 (0/19)	
3	43.0 (34/79)	55.0 (33/60)	5.3 (1/19)	
4	53.2 (42/79)	43.3 (26/60)	84.2 (16/19)	
5	2.5 (2/79)	0.0 (0/60)	10.5 (2/19)	

^a^ Subjects identified as current or prior smokers; others are non-smokers or not known. ^b^ Baseline eGFR calculated on the normalized body surface [mL/min/1.73 m^2^]. ^c^ Mono or dual antiplatelet therapy, NOAC or Phenprocoumon therapy. SD, standard deviation; BMI, Body Mass Index; CAD, coronary artery disease; MI, myocardial infarction; COPD, Chronic Obstructive Pulmonary Disease; CKD, chronic kidney disease; eGFR, Estimated Glomerular Filtration Rate; PAD, Peripheral Artery Disease; TIA, Transient Ischaemic Attack; ACT, Anticoagulation Therapy; ASA, American Society of Anesthesiologists; NOAC, New Oral Anticoagulants.

Mean age was 74.6 ± 8.2 years (median 75.0; range 60.0–89.0). Age and sex distribution were similar between the elective and emergent group (74.8 ± 7.1 vs. 73.9 ± 11.2 years; 85.0% vs. 78.9% male), with female patients accounting for 16.5% overall (15.0% elective; 21.1% emergent). Detailed demographic data and comorbidities are shown in Table 1. Cardiovascular risk factors were common: hypercholesterolemia (32.9%), hypertension (72.2%), and coronary artery disease (43.0%; including 12.7% with prior MI). Chronic kidney disease (eGFR < 60 mL/min/1.73 m^2^) was present in 34.2%—more often in emergent cases (52.6% vs. 28.3%; *p* = 0.052). One emergent patient (1.3%) had already been dialysis-dependent beforehand. Among the 19 emergent cases, 73.7% (*n* = 14) presented with free ruptured AAAs and 26.3% (*n* = 5) with acute symptomatic, but intact AAAs.

### 3.2. Aneurysm Anatomy (Table 2)

Degenerative aneurysms comprised 92.4% of cases; penetrating ulcers and inflammatory aneurysms (defined by marked periaortic inflammation and fibrosis without evidence of infection) each accounted for 3.8%. Mean maximal aortic diameter was significantly larger in emergent cases (68.3 ± 10.4 mm) than in elective repairs (57.3 ± 8.0 mm; *p* < 0.001). Proximal neck anatomy was suitable in most patients (median length 24.5 mm; diameter 22.0 mm), though 10.1% had short necks < 15 mm (emergent 26.3%; *p* = 0.007) and 10.1% had severe angulation > 60° (emergent 21.1%, *p* = 0.070), contributing to anatomical off-IFU in 20.3% of cases, with a significantly higher proportion in emergent patients (47.4% vs. 11.7%; *p* ≤ 0.001). In addition, regulatory off-IFU conditions were present in 24.1% of patients, corresponding to all 19 emergent cases. Several patients fulfilled more than one off-IFU criterion, with five patients meeting two criteria and two patients meeting three criteria. (Table 3).

**Table 2:** Anatomical characteristics among 79 patients treated with Minos stent-graft (elective: *n* = 60; emergent: *n* = 19).

Group	Total	Elective	Emergent	*p*-Value
	% (total) mean ± SD {median} [range]	% (total) mean ± SD {median} [range]	% (total) mean ± SD {median} [range]	
**Aetiology**				0.138
Aneurysm	92.4 (73/79)	93.3 (56/60)	89.5 (17/19)	
PAU	3.8 (3/79)	5.0 (3/60)	0.0 (0/19)	
Inflammatory	3.8 (3/79)	1.7 (1/60)	10.5 (2/19)	
**Aortic measurements**				
Max diameter [mm]	60.0 ± 9.8 {59.0} [40.0–83.0]	57.3 ± 8.0 {56.8} [40.0–80.0]	68.3 ± 10.4 {71.0} [51.0–83.0]	<0.001
**Aortic neck**				
Length [mm]	26.5 ± 13.1 {24.5} [3.0–78.0]	27.3 ± 12.9 {25.0} [7.0–78.0]	24.1 ± 13.6 {20.0} [3.0–54.0]	0.354
Diameter [mm]	22.7 ± 3.0 {22.0} [17.0–30.6]	22.3 ± 2.5 {22.0} [17.0–30.0]	23.8 ± 3.8 {23.9} [18.8–30.6]	0.123
Calcification **^a^** ≤25%	75.9 (60/79)	81.7 (49/60)	57.9 (11/19)	0.035
**Iliac measurements**				
**CIA diameter** **[mm]**				
Right maximum	16.6 ± 6.2 {15.7} [8.2–44.0]	16.9 ± 6.1 {16.0} [8.2–44.0]	17.0 ± 12.6 {14.0} [8.9–67.0]	0.953
Left maximum	17.0 ± 7.1 {14.6} [7.5–45.1]	16.8 ± 6.9 {14.8} [7.5–45.1]	16.1 ± 6.8 {14.4} [10.3–41.5]	0.664
**CIA length** **[mm]**				
Right maximum	66.5 ± 18.1 {64.0} [37.0–115.0]	65.8 ± 16.9 {63.0} [40.0–106.0]	78.7 ± 21.9 {66.0} [37.0–115.0]	0.543
Left maximum	67.5 ± 18.1 {66.0} [30.0–112.0]	67.1 ± 18.2 {66.0} [30.0–112.0]	78.7 ± 17.9 {68.0} [39.0–102.0]	0.728
**EIA diameter [mm]**				
Right minimum	7.2 ± 2.0 {7.0} [3.3–12.0]	7.0 ± 2.1 {7.0} [3.3–12.0]	7.6 ± 1.8 {7.5} [4.5–11.0]	0.271
Left minimum	7.1 ± 1.9 {6.8} [3.4–11.3]	7.0 ± 1.9 {6.8} [3.4–10.6]	7.3 ± 2.0 {7.3} [3.7–11.3]	0.569
**Minimum iliac access diameter**				
<7 mm	48.1 (38/79)	50.0 (30/60)	42.1 (8/19)	0.548
<5 mm	16.5 (13/79)	20.0 (12/60)	5.3 (1/19)	0.131
**Iliac tortuosity index (** **TI** **)**				
τ right	1.3 ± 0.1 {1.3} [1.1–1.7]	1.3 ± 0.1 {1.3} [1.1–1.7]	1.3 ± 0.1 {1.3} [1.1–1.6]	0.673
τ left	1.3 ± 0.1 {1.3} [1.0–1.8]	1. 3 ± 0.1 {1.3} [1.0–1.8]	1.3 ± 0.1 {1.3} [1.1–1.5]	0.804
**CIA calcification ^b^**				
**Right**				0.454
<25%	89.9 (71/79)	90.0 (54/60)	89.5 (17/19)	
25–50%	10.1 (8/79)	10.0 (6/60)	10.5 (2/19)	
**Left**				0.240
<25%	86.1 (68/79)	83.3 (50/60)	94.7 (18/19)	
25–50%	13.9 (11/79)	16.7 (10/60)	5.3 (1/19)	
**EIA calcification ^c^**				
**Right**				0.551
<25%	91.1 (72/79)	91.7 (55/60)	89.5 (17/19)	
25–50%	7.6 (6/79)	6.7 (4/60)	10.5 (2/19)	
>50%	1.3 (1/79)	1.7 (1/60)	0.0 (0/19)	
**Left**				0.811
<25%	93.7 (74/79)	93.3 (56/60)	94.7 (18/19)	
25–50%	5.1 (4/79)	5.0 (3/60)	5.3 (1/19)	
>50%	1.3 (1/79)	1.7 (1/60)	0.0 (0/19)	

^a^ Calcification or thrombus >2 mm, measured below the most distal renal artery and defined as the percentage of the circumference calcified or covered with thrombus. ^b^ Calcification or thrombus >2 mm, measured from aortic bifurcation to iliac bifurcation and defined as the percentage of the circumference calcified or covered with thrombus. ^c^ Calcification or thrombus >2 mm measured from iliac bifurcation to common femoral artery and defined as the percentage of the circumference calcified or covered with thrombus. SD, standard deviation; PAU, Penetrating Atherosclerotic Ulcer; CIA, common iliac artery; EIA, external iliac artery; TI, iliac tortuosity index.

**Table 3:** Perioperative off-IFU categorisation among 79 patients treated with Minos stent-graft (elective: *n* = 60; emergent: *n* = 19).

Group	Total	Elective	Emergent	*p*-Value
	% (total)	% (total)	% (total)	
**Anatomical off-IFU**	20.3 (16/79)	11.7 (7/60)	47.4 (9/19)	<0.001
Angulated neck >60°	10.1 (8/79)	6.7 (4/60)	21.1 (4/19)	0.070
Neck length <15 mm	10.1 (8/79)	5.0 (3/60)	26.3 (5/19)	0.007
**Regulatory off-IFU**	24.1 (19/79)	0.0 (0/60)	100.0 (19/19)	<0.001
Symptomatic but intact AAA	6.3 (5/79)	0.0 (0/60)	26.3 (5/19)	<0.001
Free rupture	17.7 (14/79)	0.0 (0/60)	73.3 (14/19)	<0.001

IFU, instructions for use; AAA, abdominal aortic aneurysms.

The majority (75.9%) showed mural thrombus or calcification less than 25% of the proximal aortic neck circumference. Distal sealing zones were generally available in the common iliac arteries (CIA) (mean diameters: right 16.6 ± 6.2 mm, left 17.0 ± 7.1 mm; lengths ~67 mm), with no relevant differences between elective and emergent cases. Calcification or thrombus < 25% of the circumference was present in 89.9% of right and 86.1% of left CIAs. External iliac arteries (EIA) had a minimum diameter of approximately 7 mm bilaterally, with >90% exhibiting calcification or thrombus <25% of the circumference. Reduced iliac access diameter <7 mm was present in 48.1% of patients (50.0% elective; 42.1% emergent; *p* = 0.548), including 16.5% with diameters <5 mm (20.0% elective; 5.3% emergent; *p* = 0.131). Adjunctive techniques (e.g., iliac angioplasty or stenting) were used selectively.

The TI was comparable across groups. Based on categorical analysis, the majority of measurements fell within category 1 (77.2%), indicating mild to moderate tortuosity, whereas only a small proportion showed higher tortuosity (7.6% for τ > 1.5), and very few exhibited severe tortuosity (2.5% for τ > 1.6). No relevant differences in the distribution of tortuosity categories were observed between elective and emergent cases (right *p* = 0.673; left *p* = 0.804).

### 3.3. Intraoperative Data (Table 4)

The Minos device was applied in all 79 included patients, without exclusion based on anatomical constraints, such as access vessel dimensions or aortic and iliac morphology. General anaesthesia was used in 72.2% (elective: 81.7%) of cases, reflecting institutional practice and shared decision making, with many patients preferring general anaesthesia for greater comfort; monitored anaesthesia care was preferred in emergent cases (57.9%) to maintain hemodynamic stability. Percutaneous access was achieved in 89.9%. Surgical cutdown was required in 10.1% of patients due to access-related issues, including significant calcification or failed preclosing; these events were considered access-related complications and did not affect technical success.

**Table 4:** Perioperative characteristics among 79 patients treated with Minos stent-graft (elective: *n* = 60; emergent: *n* = 19).

Group	Total	Elective	Emergent	*p*-Value
	% (total) mean ± SD {median} [range]	% (total) mean ± SD {median} [range]	% (total) mean ± SD {median} [range]	
**Form of anaesthesia**				<0.001
General	72.2 (57/79)	81.7 (49/60)	42.1 (8/19)	
Monitored	27.8 (22/79)	18.3 (11/60)	57.9 (11/19)	
**Operative time [min]**	122.0 ± 72.0 {110.0} [36.0–553.0]	122.5 ± 76.6 {106.0} [37.0–553.0]	118.4 ± 57.0 {112.0} [36.0–260.0]	0.829
**Fluoroscopy time [min]**	28.0 ± 18.0 {24.0} [7.0–82.0]	28.0 ± 18.0 {24.0} [7.0–82.0]	28.0 ± 19.0 {24.0} [7.0–82.0]	0.875
**Absorbed dose [mGy]**	810.3 ± 721.9 {575.8} [102.0–4645.0]	702.1 ± 537.4 {509.7} [102.0–2239.8]	1140.5 ± 1064.2 {787.0} [178.0–4645.0]	0.099
**Access**				0.721
Percutaneous	89.9 (71/79)	88.3 (53/60)	94.7 (18/19)	
Cutdown	10.1 (8/79)	11.7 (7/60)	5.3 (1/19)	
**Adjunctive procedures**				0.748
Iliac PTA/Stent **^a^**	6.3 (5/79)	6.7 (4/60)	5.3 (1/19)	
Coil embolization **^b^**	12.7 (10/79)	15.0 (9/60)	5.3 (1/19)	
Endo anchoring	8.9 (7/79)	6.7 (4/60)	15.8 (3/19)	
Proximal cuff	3.8 (3/79)	5.0 (3/60)	0.0 (0/19)	
Palmaz stent	2.5 (2/79)	1.7 (1/60)	5.3 (1/19)	
Iliac relining **^c^**	21.5 (17/79)	21.7 (13/60)	21.1 (4/19)	
IBD right/left	5.1 (4/79)	5.0 (3/60)	5.3 (1/19)	
Patch angioplasty	2.5 (2/79)	3.3 (2/60)	0.0 (0/19)	
Crossover bypass	1.3 (1/79)	0.0 (0/60)	5.3 (1/19)	
REBOA	1.3 (1/79)	0.0 (0/60)	5.3 (1/19)	
**Intraoperative death**	0.0 (0/79)	0.0 (0/60)	0.0 (0/19)	
**Length of stay [days]**	6.8 ± 3.9 {5.0} [3.0–24.0]	5.9 ± 3.0 {5.0} [3.0–16.0]	9.4 ± 5.2 {9.0} [3.0–24.0]	0.012
**ICU/IMC stay [days]**	1.7 ± 3.3 {0.0} [0.0–16.0]	0.4 ± 0.8 {0.0} [0.0–4.0]	5.9 ± 4.4 {4.0} [0.0–16.0]	<0.001
**Technical success**	93.7 (74/79)	95.0 (57/60)	89.5 (17/19)	0.389
**Clinical success**	88.6 (70/79)	90.0 (54/60)	84.2 (16/19)	0.827

^a^ PTA and/or stenting of the common iliac arteries right/left before EVAR. ^b^ Coil embolisation to prevent type II endoleak (lumbar artery, internal iliac artery, inferior mesenteric artery, renal pole artery, median sacral artery, superior gluteal artery). ^c^ Relining of aortic bifurcation or single limb graft (right/left). SD, standard deviation; PTA, percutaneous transluminal angioplasty; IBD, iliac branched device; REBOA, Resuscitative Endovascular Balloon Occlusion of the Aorta; ICU, intensive care unit; IMC, intermediate care.

Mean operative time was 122.0 ± 72.0 min (elective: 122.5 ± 76.6 min; emergent: 118.4 ± 57.0 min; *p* = 0.829). Fluoroscopy time averaged 28.0 ± 18.0 min. Radiation dose was higher in emergent cases (787.0 mGy vs. 509.7 mGy; *p* = 0.099).

**Figure 1:**
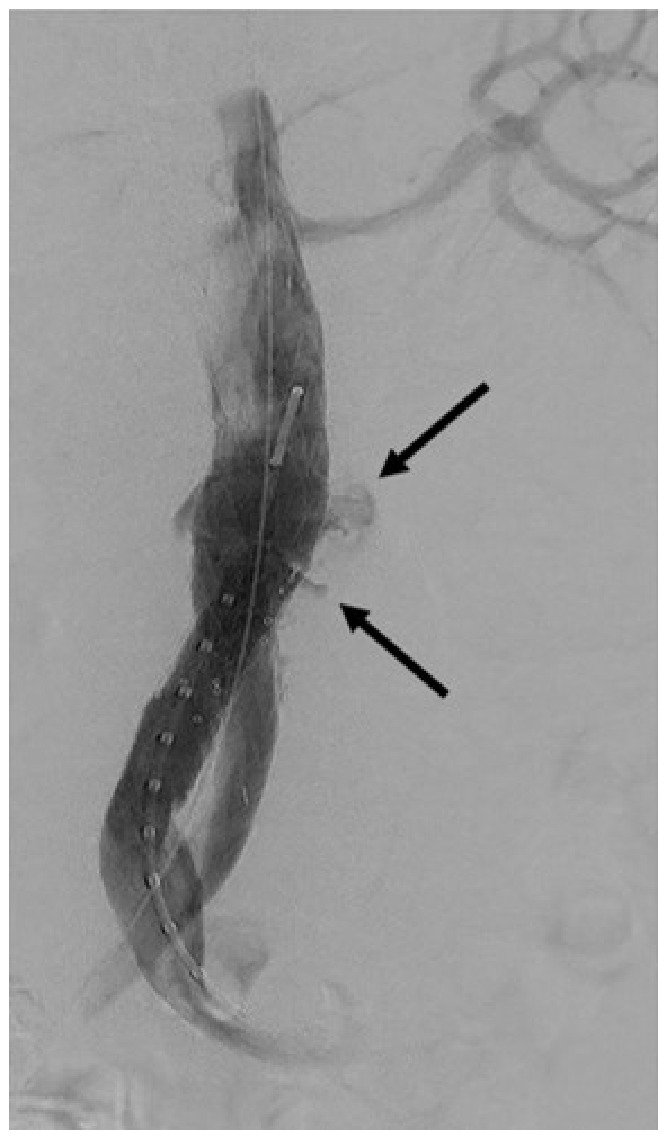
Intraoperative completion angiography demonstrating type IV endoleak (black arrows) during elective EVAR, most likely related to graft porosity of ultra-low-profile Minos stent-graft. On follow-up imaging, all but one type IV endoleaks resolved within 30 days. Remaining transient type IV endoleak was no longer detectable by postoperative day 40.

During the index procedure, one iliac limb stenosis (1.3%), three stent-graft dislocations (3.8%) and eight type I/III endoleaks (10.1%) were detected. Table 5 shows reported periprocedural endoleaks; type Ia endoleaks (6.3%) were treated with additional angioplasty or suprarenal fixation (endoanchors, cuffs, Palmaz stents). Two type Ib endoleaks (2.5%) were initially left untreated, as only minimal distal contrast leakage was observed on completion angiography despite adequate iliac sealing length and correct limb positioning, without evidence of a clinically relevant high-flow endoleak. These cases were therefore managed conservatively with close imaging surveillance, whereas all other clinically relevant endoleaks were treated accordingly. One complex type III endoleak required multiple adjuncts. Type II endoleaks occurred in 22.8%; one ruptured case required laparotomy with hematoma evacuation due to abdominal compartment syndrome and lumbar artery suturing. Four Type IV endoleaks (5.1%), in the form of oozing from the graft fabric, were detected in the immediate periprocedural phase (Figure 1). A slightly higher early incidence was noted in the elective EVAR group (6.7%), whereas no Type IV endoleaks occurred following emergent procedures. One case remained undetermined, and two cases showed more than one endoleak.

Perioperative adjunctive maneuvers were performed when clinically indicated. Iliac reinforcement was performed in 21.5%, primarily for tight bifurcations. Pre-emptive coil embolization was considered in cases with a patent inferior mesenteric artery ≥3 mm or multiple sizeable lumbar arteries on preoperative imaging, and was performed in 12.7% of patients. Endoanchors were applied in 8.9% of cases, more frequently in emergent cases (15.8%). Proximal cuffs and Palmaz stents were used in 3.8% and 2.5%, respectively. No intraoperative ruptures or deaths occurred.

### 3.4. Thirty-Day Outcomes

Mean hospital stay was 6.8 ± 3.9 days (elective 5.9 ± 3.0; emergent 9.4 ± 5.2; *p* = 0.012). Intensive care unit (ICU)/intermediate care monitoring was brief after elective EVAR (0.4 ± 0.8 days) but was significantly prolonged in emergent cases (5.9 ± 4.4 days; *p* < 0.001). Length of stay reflects total in-hospital duration and may include preoperative waiting time, as separate postoperative data were not systematically recorded. 30-day mortality was 2.5% (2/79); both patients had ruptured AAAs. One death was due to hypoxic brain injury following massive pulmonary embolism on POD 12, and the second occurred on POD 12 after transfer to an external hospital, with the cause remaining unknown. In addition, two further patients (2.5%) did not attend the scheduled 30-day follow-up and were considered lost to follow-up.

Table 6 summarizes perioperative complications within 30 days post-intervention. MI occurred in 10.5% of emergent cases versus none in the elective group (*p* = 0.056), all classified as Non-ST-Elevation Myocardial Infarction (NSTEMI). One cardiac arrest occurred in the emergent group (5.3%, *p* = 0.241). Hospital-acquired pneumonia was present in 31.6% of emergent patients and absent in elective cases (*p* < 0.001). Respiratory failure, spinal cord ischemia, and sepsis each occurred in two patients (10.5%) in the emergent group (*n* = 19), whereas none were observed in the elective group. Notably, both cases of spinal cord ischemia were transient and resolved completely without permanent neurological deficit. Acute kidney failure occurred in 31.6% of emergent and 3.3% elective cases (*p* = 0.004), with two requiring dialysis (all emergent: one transient, one permanent). Post-implantation syndrome was more common in elective cases (25.0% vs. 5.3%, *p* = 0.062). Other complications (stroke, bowel ischemia, graft thrombosis, limb occlusion) showed no significant differences.

During 30-day follow-up, 13 of 79 patients (16.5%) required reintervention. Rates were significantly higher in emergent cases (31.6%) compared with elective procedures (11.7%, *p* = 0.041). In elective patients, early reinterventions were predominantly related to access-site complications, such as wound revision, pseudoaneurysm, or hematoma. In contrast, reinterventions in emergent cases were primarily aneurysm-related, including abdominal compartment syndrome requiring hematoma evacuation and persistent endoleaks.

Initial imaging confirmed successful aneurysm exclusion in most cases (Table 5); type I endoleaks occurred primarily in ruptured cases (10.5%). One type Ia endoleak was left untreated due to severe respiratory failure. One complex type Ib endoleak was managed by coil embolization of the left iliac limb and femoral–femoral crossover bypass to maintain limb perfusion in the context of a suspected free rupture, necessitating immediate and definitive exclusion of any potential bleeding source. One type III endoleak was managed with iliac relining and cuff implantation. Type II endoleaks were the most common (25.3%) and were typically managed conservatively. Type IV endoleaks were observed only in the early postoperative phase and resolved spontaneously. They occurred in 1.3% in the elective group within 30 days, with no cases beyond that period. No type V endoleaks were observed.

### 3.5. Early Observations

**Figure 2:**
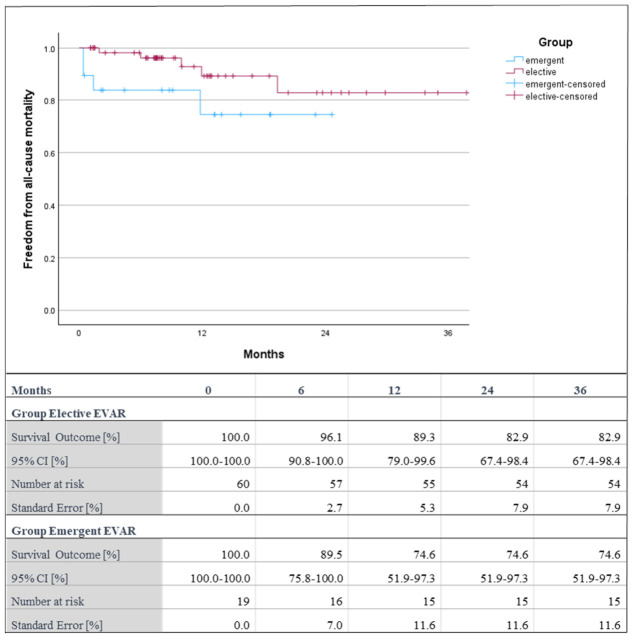
Kaplan–Meier survival plot showing freedom from all-cause mortality in elective (red) and emergent (blue) EVAR cases. Censored observations are indicated by tick marks. Survival estimates with corresponding 95% confidence intervals and numbers at risk are provided in table below. Survival between groups was compared using log-rank test (*p* = 0.100). Estimates are shown up to 36 months.

Mean follow-up was 12.5 ± 9.9 months (median 9.3; range 0.4–41.8 months). Six-month survival was 96.1% (95% CI 90.8–100%) in the elective cohort and 89.5% (95% CI 75.8–100%) in the emergent cohort (Figure 2). At 12 months, survival declined to 89.3% (95% CI 79.0–99.6%) and 74.6% (95% CI 51.9–97.3%), respectively, remaining stable within the observed follow-up period. No significant difference in survival between groups was observed (log-rank *p* = 0.100). Between 30 days and study end, 8/77 patients died (10.4%): six elective (7.8%) and two emergent (2.6%). No aneurysm-related deaths occurred in the further follow-up period. One patient with advanced peripheral arterial disease died from cardiac and renal failure following bilateral lower limb ischemia and rhabdomyolysis. Imaging showed no endograft thrombosis or device dysfunction; a device-related cause was considered unlikely, although the exact mechanism remained unclear. Three deaths were due to respiratory failure (pulmonary fibrosis or pneumonia), one from necrotizing pancreatitis and three were of unknown cause. At the end of the observation period, overall survival remained 82.9% in the elective group and 74.6% in the emergent group.

Freedom from aneurysm-related reintervention beyond 30 days post-procedure was 94.8% (elective 93.3% vs. emergent 100.0%; *p* = 0.194). Over the entire observation period, a total of 24 reinterventions were performed in 22 patients (27.8%), with two patients undergoing more than one procedure. Beyond 30 days, an additional 11 secondary interventions were performed. Aneurysm- or device-related reinterventions accounted for five cases (5/24; 20.8%), including two limb occlusions (8.3%) requiring thrombectomy and relining and three kink-related iliac limb stenoses (8.3%) treated with stent reinforcement. Access-related reinterventions were primarily confined to the early postoperative period. The remaining procedures were unrelated to the index EVAR and addressed other vascular conditions, including thoracic endovascular aortic repair, thoracoabdominal endografting, femoropopliteal bypass, and renal artery stenting. All Minos main bodies remained patent, and no stent-graft infection was observed.

Aneurysm sac regression (≥5 mm) was observed in 43.0% of patients—41.7% elective and 47.4% emergent cases (*p* = 0.662). Stable sac size was seen in 55.7%, and only 1.3% showed sac growth >5 mm (persistent endoleak Ia), which was treated with a custom-made fenestrated graft.

Imaging outcomes (Table 5) revealed late endoleaks (>30 days). Among 18 initial type II endoleaks (22.8%), 44.4% sealed spontaneously; 10 persisted (10.0% elective, 21.1% emergent, *p* = 0.207), of which most showed no sac progression and were further monitored. Of seven perioperative type I endoleaks (8.9%), all but one resolved. One type III endoleak resolved by POD 42; one primary type IV endoleak was undetectable by POD 40.

Technical and clinical success rates were high in both groups, with no statistically significant difference. Overall technical success was 93.7%, with rates of 95.0% in elective and 89.5% in emergent cases (*p* = 0.389), while overall clinical success reached 90.0% in the elective cohort and 84.2% in the emergent cohort (*p* = 0.827).

## 4. Discussion

The Minos stent-graft was evaluated in elective and emergent EVAR, focusing on anatomical complexity, perioperative risk, and early durability. Minos demonstrated consistent technical performance across both settings, with early outcome differences attributable to the higher clinical complexity of emergency cases rather than to device-related factors. Findings indicate that Minos is well-suited for a patient population facing real-world challenges—often elderly, comorbid, and anatomically off-IFU.

When interpreting the results of the present study, a descriptive overview across predefined outcome domains is provided to place the findings for the Minos stent-graft within the context of the existing literature. The current cohort comprised a substantial proportion of anatomically complex cases, including hostile proximal neck anatomy and decreased iliac diameter. These features have been associated with an increased risk of technical failure, endoleaks, and reintervention [29]. Overall, 20.3% of patients were treated outside anatomical IFU, with a significantly higher proportion in emergent cases (47.4% vs. 11.7%, *p* ≤ 0.001). This may have contributed to increased procedural complexity, reflected by prolonged fluoroscopy times (mean 28.0 ± 18.0 min), as well as to the overall reintervention rate of 27.8%.

In this context, a high proportion of patients in the present study also exhibited reduced iliac access diameters, with 48.1% presenting with <7 mm (50.0% elective; 42.1% emergent; *p* = 0.548), including 16.5% with <5 mm (20.0% elective; 5.3% emergent; *p* = 0.131). The low-profile design of the Minos device (14F main body, corresponding to an access vessel requirement of approximately ≥5 mm) may therefore facilitate treatment in patients with limited access diameters. Iliac tortuosity was comparable across groups, with the majority of patients exhibiting mild to moderate tortuosity (77.2%), while higher degrees of tortuosity were observed in only a small proportion of cases, suggesting that access vessel morphology was unlikely to have influenced procedural differences or outcomes.

The proportion of female patients (15.0% elective; 21.1% emergent) was consistent with previously reported EVAR cohorts, in which women typically account for up to 25% of treated patients [30]. Despite this, female patients remain underrepresented in EVAR populations, largely due to anatomical constraints, such as smaller iliac access vessels and more complex aortoiliac anatomy, which may limit eligibility for standard devices. In addition, sex-specific differences in aneurysm biology, including an increased risk of rupture at smaller diameters, may influence clinical presentation. Moreover, women have been shown to experience worse outcomes after EVAR, with meta-analytic data demonstrating a significantly increased risk of perioperative mortality (30-day mortality OR 1.67; in-hospital mortality OR 1.90), as well as higher rates of limb ischemia (OR 2.44) and other complications compared with men [31,32]. In this context, the use of low-profile devices may facilitate endovascular treatment and help expand EVAR eligibility in this subgroup.

However, EVAR was performed in all cases, with no intraoperative death or device-related conversion (30-day mortality 2.5%). The observed incidence of spinal cord ischemia in the emergent group (10.5%) may partly reflect the critical clinical condition of these patients and the small sample size, rather than necessarily indicating an increased complication risk. Previous clinical studies evaluating Minos included elective, emergent, ruptured, and off-IFU cases, although the proportion of hostile anatomy and urgency of repair differed across study populations [19,20,21].

Overall technical success was 93.7%, with a clinical success rate of 88.6%. Published Minos series reported technical success rates of approximately 95% in elective populations [19] and high procedural success in mixed elective and emergent cohorts [20,21]. High technical success has also been reported for other contemporary EVAR systems, including 99.3% for Cook Zenith Alpha [33], while large Endurant series describe high procedural success across elective and emergent indications [34]. Interpretation of these figures should account for differences in anatomical inclusion criteria, urgency of repair, and study design.

Regarding endoleak behaviour, low rates of persistent high-grade endoleaks were observed in the present Minos cohort, with type Ia endoleaks present in 1.3% of patients at follow-up and no type III endoleaks detected. Although type I endoleaks were frequently identified intraoperatively (8.9%), they were successfully treated during the index procedure, resulting in effective aneurysm exclusion in most cases. In selected cases, including type Ib endoleaks identified at completion angiography during the index procedure, only minimal residual contrast leakage, without evidence of a relevant high-flow endoleak, was observed and therefore managed conservatively with close imaging surveillance, which is in line with previous reports describing spontaneous resolution of selected type I endoleaks within weeks after EVAR [35]. Other Minos studies primarily addressed type I and III endoleaks and reported no relevant high-grade endoleaks and no device migration during follow-up [19,20,21]. For other EVAR platforms, long-term data for the Gore Excluder report persistent type Ia endoleaks in approximately 4.8% and type Ib endoleaks in 2.8% of cases [36], while INCRAFT series documented type III endoleaks of approximately 4% and secondary open conversion rates of up to 5% [37]. Reported endoleak rates should therefore be interpreted in the context of heterogeneous follow-up durations and reporting standards.

Sac behaviour was assessed as a surrogate marker for successful aneurysm exclusion and long-term durability [38,39]. In the present cohort, sac regression ≥5 mm was observed in 43.0% of patients at a mean follow-up of 12.5 months. Sac growth exceeding 5 mm was observed in 1.3% and was attributed to a persistent type Ia endoleak, managed with a custom-made fenestrated graft. Higher rates of sac regression (approximately 70%) have been reported for Minos at longer follow-up durations (mean 30 months) in elective cohorts [19], while other Minos studies described stable sac dimensions without late expansion in both standard and hostile anatomy [20,21]. Differences in reported sac dynamics may reflect variation in follow-up duration, baseline aneurysm morphology, and anatomical complexity. Long-term sac stabilisation has also been documented for Endurant and Excluder systems, predominantly at follow-up intervals exceeding 3 years [34,36].

Limb-related complications remain an important consideration for EVAR. In the present Minos cohort, iliac limb graft occlusion occurred in 2.6% of patients at a mean follow-up of 12.5 months. For the Zenith Alpha endograft, limb-related complication rates ranging from 7% to 15% have been reported at approximately 3 years postoperatively [17,40], while limb thrombosis and intraprosthetic thrombus formation have also been described for Endurant II with comparable follow-up durations (5% beyond 2–3 years) [40].

To prevent iliac limb occlusion, prophylactic iliac reinforcement during EVAR may be considered [41]. At the treating centre, this approach is routinely implemented in patients with a distal aortic bifurcation <20 mm, irrespective of graft type or manufacturer, and was applied in 21.5% of cases. A bifurcation diameter of ≤20 mm has been described in the literature as a threshold for a narrow aortic bifurcation and is associated with an increased risk of limb compression and occlusion, which may justify this approach in selected cases [42]. Published long-term limb patency data for Minos remain limited, and extended follow-up of the present cohort may provide additional descriptive information.

Late reintervention occurred in 13.9% of patients treated with Minos. Published Minos cohorts reported variable device-related reintervention rates depending on follow-up duration and endpoint definitions [19,20,21]. For Endurant, cumulative reintervention rates of approximately 17.4% have been reported over longer observation periods [34], while Gore Excluder cohorts describe ongoing secondary interventions during surveillance despite durable aneurysm exclusion [36]. Higher reintervention and conversion rates have been reported for INCRAFT (31%) [37]. Direct numerical comparison across devices remains limited by heterogeneity in study design, reporting standards, and follow-up duration.

An additional observation of this study relates to the occurrence of type IV endoleaks in the setting of ultra-low-profile stent-grafts. Type IV endoleaks were identified in 6.7% of elective cases. No type IV endoleak was observed beyond 30 days after the index procedure and no secondary interventions were required. Additionally, follow-up imaging demonstrated neither aneurysm sac expansion nor rupture. As ultra-low-profile stent-grafts are increasingly used in complex EVAR, careful evaluation of low-grade endoleak behaviour is warranted, as device design, graft material characteristics, and deployment may influence both their incidence and detection. In this context, type IV endoleaks may be clinically relevant in selected patient populations, including those with coagulopathy, large aneurysm sacs, or ruptured aneurysms. Prior studies evaluating Minos predominantly focused on high-grade endoleaks (type I and III), whereas type IV endoleaks were either not reported or not analysed separately [19,20,21]. The present findings add descriptive data on the incidence and short- to mid-term behaviour of type IV endoleaks following Minos implantation and highlight the need for systematic documentation and postoperative surveillance as device design increasingly prioritizes deliverability while maintaining structural durability.

### Limitations

This retrospective single-centre study—especially within the emergent subgroup—is limited by its small sample size and associated risk of selection bias, reducing the generalisability of the findings. With only 19 emergent patients, the study is likely underpowered to detect rare adverse events, such as rupture or conversion, weakening comparative conclusions. The absence of a comparator cohort further prevents assessment of the relative efficacy of the Minos stent-graft. Incomplete imaging follow-up due to death, refusal, or loss to follow-up may have led to underreporting of endoleaks or sac dynamics. Moreover, the maximum observation period of 41.8 months restricts evaluation of late complications, as device-related failures may occur beyond 2–5 years, introducing potential attrition bias. These limitations highlight the need for prospective multi-centre studies with standardised imaging and extended long-term follow-up to validate these results.

## 5. Conclusions

As the vascular population ages, more complex anatomies and higher perioperative risk increase the need for deliverable and reliable EVAR systems. Ultra-low-profile devices, such as Minos, may facilitate endovascular treatment in patients with challenging access anatomy and may potentially expand EVAR eligibility in selected cases. Clinical experience across elective, emergent, ruptured, and off-IFU cases shows high technical success, low reintervention rates, and consistent sac regression in the present cohort. Prior European Minos series have additionally confirmed durability with low rates of high-risk endoleaks (type I/III) and no device migration during follow-up; in the present study, type IV endoleaks occurred but had no clinical impact. The ability to treat ruptured AAAs percutaneously with minimal adjuncts may further reduce ICU stay and morbidity with potential cost benefits. Together, these findings indicate that ultra-low-profile systems may expand treatment options for high-risk patients, although long-term multi-centre data remain necessary to validate these early results.

## Figures and Tables

**Table 5:** Summary of reported endoleaks among 79 patients treated with Minos stent-graft (elective: *n* = 60; emergent: *n* = 19).

Time Point	Index Procedure	≤30 Days Postprocedural	>30 Days Postprocedural
**Total EVAR**			
EL I	8.9 (7/79)	2.5 (2/79)	1.3 (1/79)
Ia	6.3 (5/79)	1.3 (1/79)	1.3 (1/79)
Ib	2.5 (2/79)	1.3 (1/79)	0.0 (0/79)
EL II	22.8 (18/79)	25.3 (20/79)	12.7 (10/79)
EL III	1.3 (1/79)	1.3 (1/79)	0.0 (0/79)
EL IV	5.1 (4/79)	1.3 (1/79)	0.0 (0/79)
EL V	0.0 (0/79)	0.0 (0/79)	0.0 (0/79)
Undetermined	1.3 (1/79)	0.0 (0/79)	0.0 (0/79)
**Elective EVAR**			
EL I	10.0 (5/60)	0.0 (0/60)	1.7 (1/60)
Ia	5.0 (3/60)	0.0 (0/60)	1.7 (1/60)
Ib	3.3 (2/60)	0.0 (0/60)	0.0 (0/60)
EL II	25.0 (15/60)	16.7 (10/60)	10.0 (6/60)
EL III	0.0 (0/60)	1.7 (1/60)	0.0 (0/60)
EL IV	6.7 (4/60)	1.7 (1/60)	0.0 (0/60)
EL V	0.0 (0/60)	0.0 (0/60)	0.0 (0/60)
Undetermined	0.0 (0/60)	0.0 (0/60)	0.0 (0/60)
**Emergent EVAR**			
EL I	15.8 (3/19)	10.5 (2/19)	0.0 (0/19)
Ia	10.5 (2/19)	5.3 (1/19)	0.0 (0/19)
Ib	0.0 (0/19)	5.3 (1/19)	0.0 (0/19)
EL II	15.8 (3/19)	52.6 (10/19)	21.1 (4/19)
EL III	5.3 (1/19)	0.0 (0/19)	0.0 (0/19)
EL IV	0.0 (0/19)	0.0 (0/19)	0.0 (0/19)
EL V	0.0 (0/19)	0.0 (0/19)	0.0 (0/19)
Undetermined	5.3 (1/19)	0.0 (0/19)	0.0 (0/19)

Percentages are reported based on total cohort at each time point; deceased patients were not excluded from denominator. EVAR, endovascular aneurysm repair; EL, endoleak.

**Table 6:** Perioperative complications within 30 days among 79 patients treated with Minos stent-graft (elective: *n* = 60; emergent: *n* = 19).

Group	Total	Elective	Emergent	*p*-Value
	% (total) mean ± SD {median} [range]	% (total) mean ± SD {median} [range]	% (total) mean ± SD {median} [range]	
**MI**	2.5 (2/79)	0.0 (0/60)	10.5 (2/19)	0.056
STEMI	0.0 (0/79)	0.0 (0/60)	0.0 (0/19)	
NSTEMI	2.5 (2/79)	0.0 (0/60)	10.5 (2/19)	
**Cardiac arrest**	1.3 (1/79)	0.0 (0/60)	5.3 (1/19)	0.241
**HAP**	7.6 (6/79)	0.0 (0/60)	31.6 (6/19)	<0.001
Respiratory failure	2.5 (2/79)	0.0 (0/60)	10.5 (2/19)	0.011
**Stroke**	1.3 (1/79)	0.0 (0/60)	5.3 (1/19)	0.074
**Spinal cord ischemia**	2.5 (2/79)	0.0 (0/60)	10.5 (2/19)	0.011
Transient	2.5 (2/79)	0.0 (0/60)	10.5 (2/19)	
Paraplegia	0.0 (0/79)	0.0 (0/60)	0.0 (0/19)	
**Acute kidney failure**	10.1 (8/79)	3.3 (2/60)	31.6 (6/19)	0.004
Permanent dialysis	1.3 (1/79)	0.0 (0/60)	5.3 (1/19)	
**Bowel ischemia**	0.0 (0/79)	0.0 (0/60)	0.0 (0/19)	
**Sepsis**	2.5 (2/79)	0.0 (0/60)	10.5 (2/19)	0.011
**Limb occlusion**	0.0 (0/79)	0.0 (0/60)	0.0 (0/19)	
**Post-implantation syndrome**	20.3 (16/79)	25.0 (15/60)	5.3 (1/19)	0.062
**Graft infection**	0.0 (0/79)	0.0 (0/60)	0.0 (0/19)	
**Graft thrombosis**	10.1 (8/79)	11.7 (7/60)	5.3 (1/19)	0.420
≤25%	10.1 (8/79)	11.7 (7/60)	5.3 (1/19)	
>25%	0.0 (0/79)	0.0 (0/60)	0.0 (0/19)	
**Aneurysm rupture**	0.0 (0/79)	0.0 (0/60)	0.0 (0/19)	
**Reintervention**	16.5 (13/79)	11.7 (7/60)	31.6 (6/19)	0.041
**Vascular access complication**	10.1 (8/79)	11.7 (7/60)	5.3 (1/19)	0.420

SD, standard deviation; MI, myocardial infarction; STEMI, ST-elevation myocardial infarction; NSTEMI, Non-ST-elevation myocardial infarction; HAP, hospital-acquired pneumonia.

## Data Availability

The data supporting the findings of this study are available from the corresponding author upon reasonable request. The data are not publicly available due to privacy and ethical restrictions.

## Abbreviations

The following abbreviations are used in this manuscript:

AAA	Abdominal aortic aneurysm
ACT	Anticoagulation Therapy
ASA	American Society of Anesthesiologists
BMI	Body Mass Index
CAD	Coronary artery disease
CEUS	Contrast-enhanced ultrasound
CIA	Common iliac artery
CKD	Chronic kidney disease
COPD	Chronic Obstructive Pulmonary Disease
CTA	Computed Tomography Angiography
eGFR	Estimated Glomerular Filtration Rate
EIA	External iliac artery
EL	Endoleak
EVAR	Endovascular aneurysm repair
Fr	French
HAP	Hospital-acquired pneumonia
IBD	Iliac branched device
ICU	Intensive care unit
IFU	Instructions for use
IMC	Intermediate care
MAE	Major adverse event
MI	Myocardial infarction
NOAC	New Oral Anticoagulants
NSTEMI	Non-ST-Elevation Myocardial Infarction
PAD	Peripheral Artery Disease
POD	Postoperative Day
PTA	Percutaneous transluminal angioplasty
REBOA	Resuscitative Endovascular Balloon Occlusion of the Aorta
SD	Standard deviation
STEMI	ST-elevation myocardial infarction
SVS	Society for Vascular Surgery
TI	Iliac tortuosity index
TIA	Transient Ischaemic Attack
